# Early‐life blood pressure and midlife brain and cognitive health: tests in two birth cohorts

**DOI:** 10.1002/alz70860_100740

**Published:** 2025-12-23

**Authors:** Mayibongwe Mugoba, Scott T Chiesa, Renate Houts, Annchen R. Knodt, Reremoana Theodore, Ahmad R. Hariri, Avshalom Caspi, Terrie E. Moffitt

**Affiliations:** ^1^ University College London, London, England, United Kingdom; ^2^ UCL Unit for Lifelong Health and Ageing, London, England, United Kingdom; ^3^ Duke University, Durham, NC, USA; ^4^ University of Otago, Dunedin, Otago, New Zealand; ^5^ King's College London, London, United Kingdom

## Abstract

**Background:**

Elevated blood pressure (BP) in midlife is a well‐established risk factor for impaired brain health and cognitive ability in old age. We hypothesised that exposure to elevated BP within the first five decades of life may contribute to this risk through impacts on brain health and/or cognitive ability evident by midlife.

**Method:**

Participants were selected from the Dunedin Multidisciplinary Health and Development Study (*n* = 893). Exposures were systolic (SBP) and diastolic (DBP) blood pressures measured at ages 7, 11, 18, 26, 32, 38 and 45. Cumulative early‐life exposure to blood pressure was also quantified as area under the curve (AUC). Brain health was assessed at age 45 via imaging measures comprising BrainAge, white matter hyperintensity (WMH) burden, and retinal arteriolar calibers (RAC) ‐ a proxy for cerebral small vessel remodeling. Cognitive ability (IQ) was also measured at age 45, with replication of cognitive findings tested in a larger contemporary cohort (1970 British Cohort Study).

**Result:**

We found limited evidence for any association between BP in the first four decades of life and brain health or cognitive ability at age 45. Most associations instead emerged for BP from ∼age 40 onwards. Midlife BP was associated with older BrainAge (Beta for DBP at age 45 = 0.11 [0.04, 0.19]; *p* = 0.003) and more WMH burden (Beta = 0.09 [0.02, 0.17]; *p* = 0.019). Effect sizes for SBP were similar. For cognitive ability, DBP at ages 38 and 45 showed modest associations with age‐45 IQ, which became null after accounting for childhood IQ. These findings replicated in the British Cohort for age‐47 IQ. Only RAC were found to associate with BP from childhood (Beta for age‐7 DBP = ‐0.09 [‐0.16, ‐0.03]; *p* = 0.006), and the magnitude of these estimates increased during midlife (Beta at age 45 = ‐0.37 [‐0.45, ‐0.30]; *p* < 0.001).

**Conclusion:**

We found little evidence for any association between BP prior to age 40 and BrainAge, WMH volume, or cognitive ability in midlife. However, cumulative exposure to elevated BP from childhood was associated with reduced RAC, suggesting a potential link between BP and adverse cerebral small vessel remodeling from childhood.

